# Seabird strandings on the Brazilian coast: What influences spatial and temporal patterns?

**DOI:** 10.1371/journal.pone.0317335

**Published:** 2025-04-16

**Authors:** Regiane da Silva Rodrigues, Vivian de Mello Cionek, André Silva Barreto, Joaquim Olinto Branco

**Affiliations:** 1 Graduate Program in Ecology and Natural Resources, Federal University of São Carlos – UFSCar, São Paulo, Brazil; 2 Graduate Program in Environmental Sciences and Technology, University of Vale do Itajaí – UNIVALI, Itajaí, Santa Catarina, Brazil; 3 Biodiversity Informatics and Geomatic Laboratory (LIBGeo), University of Vale do Itajái – UNIVALI, Itajaí, Santa Catarina, Brazil; CNESTEN: Centre National de l'Energie des Sciences et des Techniques Nucleaires, MOROCCO

## Abstract

Seabirds exhibit physiological adaptations that allow them to forage in the marine environment and undertake long-distance migrations during non-reproductive periods. As a result, they face various natural and anthropogenic pressures, which can lead to extreme fatigue and even death. Stranded bodies that float in the sea can wash ashore, providing valuable ecological information. This study aimed to analyze seabird strandings along the south and southeast coasts of Brazil between 2016 and 2019, focusing on spatiotemporal and potential environmental and anthropogenic influences. Using data from the Santos Basin Beach Monitoring Project, we calculated ecological indices of abundance, richness, and diversity for the entire seabird community and separately by migratory behavior (resident, southern migratory, northern migratory). Statistical modeling revealed a strong decreasing trend in strandings from south to north, with higher events on the southern coast (Santa Catarina and Paraná) and lower on the southeast coast (São Paulo). Resident species and northern migratory species showed peak strandings in spring, while southern migratory peaked in winter. These spatial and temporal patterns reflected birds’ home ranges, reproductive cycles, and migratory behaviors. Environmental variables influenced strandings differently depending on species migration behavior and ecological indices, highlighting the role of oceanographic processes in carcass drift and the impact of climatic events on species mortality. This study is the first to demonstrate a spatiotemporal pattern of seabird strandings on the Brazilian coast, providing valuable insights into seabird dynamics in the Santos Basin and offering important data for conservation efforts.

## Introduction

Seabirds are directly influenced by both small- and large-scale environmental changes in marine ecosystems. These long-lived species—some reaching up to 60 years—exhibit low fecundity, late sexual maturity, and colonial nesting habits [1–3]. They undertake long migrations or daily movements during foraging, which are physiologically demanding. Along the way, they may encounter a range of climatic events and changes in prey availability that directly impact survival rates. Additionally, seabirds face exposure to various anthropogenic pollutants, such as plastics, heavy metals, and petroleum products [1,4–9]. The combined natural and anthropogenic pressures likely contribute to increased morbidity and mortality, though the patterns and direct drivers are multifaceted. It is common to find debilitated or dead birds floating at sea or stranded on beaches, often with no immediately identifiable cause [10–12].

Bird strandings are common on Brazilian beaches, but mass stranding events, characterized by large numbers of individuals in a short time, are rare. Although these events have been observed in southern Brazil since 1997 [13], identifying the cause of mass strandings is challenging [14,15], even when limited to a few species, such as the Procellariiformes mass stranding event during March 2013 [11]. The constant presence of carcasses on beaches may depend on animal mortality and a body’s drifting capacity [12]. Wind, wave height, and ocean currents can also influence this drift [16], though these effects are not fully understood.

Migratory species face additional stresses and limitations, as they must adjust their diets along the migration route [17]. Large-scale climate change can affect ocean productivity and disrupt prey availability [18], while storm events can cause disorientation and increased energy expenditure. Ocean currents, wind direction, and intensity, can wash these birds ashore — alive or dead [10,12,19].

Thus, stranded animal surveys can contribute to investigating the processes that lead to strandings and help identify potential patterns associated with them [20,21]. Carcasses can also be used to understand possible causes of mortality and related processes, as well as to gain insights into the ecology of the species, such as its diet and life history traits [16,22]. This is especially important given the challenges in studying many seabird species and the current knowledge gaps regarding them.

The aim of this study was to analyze the spatiotemporal distribution of the abundance, richness, and diversity of seabird species stranded along the south and southeast Brazilian coasts between 2016 and 2019. Additionally, we investigated whether ecological indices are influenced by species movement patterns, categorized as resident species, southern hemisphere migrants, and northern hemisphere migrants. Finally, we examined the potential association between environmental and anthropogenic variables and stranding patterns, offering insights into how these factors may drive the observed dynamics. To guide our investigation, we formulated the following hypotheses: (i) the abundance, richness, and diversity of seabird species are strongly influenced by seasonal periods. Resident species are expected to exhibit higher diversity, abundance, and richness during their reproductive peaks. For migratory species, these indices are expected to be higher during the seasons corresponding to their arrival along the Brazilian coast as they move away from primary breeding sites. (ii) We hypothesize that abundance, richness, and diversity, regardless of species movement patterns, will be higher under increased wave height and wind intensity, predominant northerly winds, higher sea surface temperatures, and elevated chlorophyll concentrations. These variables are expected to directly influence the corpses of seabirds washed ashore, as well as increase the presence of birds in the surveyed areas, because they serve as indirect indicators of both climatic variability and upwelling zones with higher energy availability for seabirds. Finally, (iii) we expect that vessel traffic intensity will be positively correlated with higher stranding rates, as the vessels examined in this study are associated with support operations for oil and natural gas production. These activities may influence the number of individuals affected by oil contamination, potentially increasing stranding events.

## Materials and methods

### Study area

**Fig 1:**
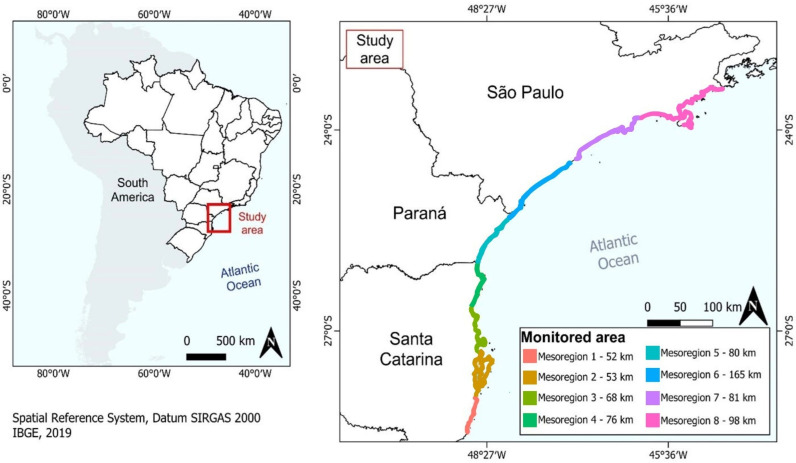
Study area. Location of the study area emphasizing the mesoregions and monitored area by the Santos Basin Beach Monitoring Program (PMP-BS). Spatial data were extracted from the IBGE dataset [25], using the QGIS software [26].

The study area comprises eight mesoregions, covering approximately 1,032.64 km of beach extension monitored daily, from the municipality of Laguna in the south of the state of Santa Catarina (28º28’57” S, 48º46’53” W) up to the municipality of Ubatuba, north of the state of São Paulo, Brazil (23º26’02” S, 45º04’15” W) (Table 1, Fig 1). Although the mesoregions are a political management categorization used in Brazil, each one has a set of beaches with very similar physiographic characteristics, that differentiate each mesoregion from one another [23].

**Table 1:** Mesoregions. Description of the mesoregions, including their identification number, the states they belong to, and their names as defined by IBGE.

Mesoregion Number	State	Name
1	Santa Catarina	South Coast of Santa Catarina
2	Santa Catarina	Central Coast of Santa Catarina
3	Santa Catarina	Central-North Coast of Santa Catarina
4	Santa Catarina	North Coast of Santa Catarina
5	Paraná	Coast of Paraná
6	São Paulo	South Coast of São Paulo
7	São Paulo	Central Coast of São Paulo
8	São Paulo	North Coast of São Paulo

The study area is an important habitat for marine species, where it is common to find not only resident seabirds but also migratory seabirds that use the beaches for resting and feeding [24]. This region is part of the Santos Basin, the largest offshore sedimentary basin in Brazil, which extends from Cabo Frio (Rio de Janeiro state) to Florianópolis (Santa Catarina state).

### Sampling

The stranded seabird data were obtained from the Santos Basin Beach Monitoring Project (PMP-BS), one of several monitoring programs required by IBAMA (Brazil’s federal environmental agency) for the environmental licensing process for Petrobras’ oil and natural gas production in the pre-salt region (an area of oil reserves located beneath a thick layer of salt in the seabed). The monitoring was conducted daily by the PMP-BS team between 2016 and 2019, and all bird strandings (live or carcasses) were systematically recorded and identified based on information in the literature, and subsequently removed from the beach to prevent recounting [20].

To test our first hypothesis we classified all stranded bird records from PMP-BS study area into three ecological groups: Seabirds, Waterbirds, and Shorebird However, we excluded Waterbirds and Shorebirds from our analysis, as these groups are primarily dependent on terrestrial habitats and resources and are not directly influenced by oceanic variables. Considering seabirds, we classified them as either resident, southern, or northern migratory species. Residents were defined as species that occupy the same area year-round, although some populations may engage in partial movements outside these areas. Migratory species were categorized based on their primary breeding sites, identifying whether they originated from the southern or northern hemisphere. The classification was made based on supporting literature [17,27,28] and is noted in Supporting Information (See S1 File). We removed *Spheniscus magellanicus* from the seabird dataset because penguins are the only aquatic bird and are not affected by environmental variables in the same way that it does for aerial species. In addition, this single species represents half of the total dataset, which would inflate the results for southern species and lead to an incorrect interpretation of the results. Owing to the importance of the strandings of this species, we conducted separate analyses, delving into the entire population structure and potential impacts [29].

To calculate the diversity indices of stranding seabirds (abundance, species richness and Hill’s number for taxonomic diversity (exp(H’), with *q = *1), we used the function ‘*diversity*’ of the ‘*vegan*’ package [30]. The diversity indices were calculated for all seabird species and separately for the three categories: Resident Seabird, Southern Migratory Seabird and Northen Migratory Seabird.

To test the second hypothesis, five environmental variables were used to assess their influence on seabird strandings: wave height (WH), sea surface temperature (SST), concentration of chlorophyll (CC), wind intensity (WI), wind direction (WD). CC and SST data comes from the AQUA satellite with resolution of 4 km/pixel and images were obtained from the page PODDAC (Physical Oceanography Distributed Active Archive Center–https://podaac.jpl.nasa.gov/). WH, WI and WD data were obtained using a model called WAVEWATCH III ®, developed by NOAA/NCEP (http://polar.ncep.noaa.gov/waves/viewer.shtml). These data were available every three hours. To test the third hypothesis, two anthropogenic variables were measured to better understand the potential impact of human pressures, such as oil contamination, on seabird strandings: the intensity of Vessel traffic operated by Petrobras (VTP), consisting of large support vessels used in the oil and natural gas production process by Petrobras, and the intensity of other traffic vessels (OTV) not related to oil activities. The VTP and OTV were provided by the Vessel Traffic Monitoring Project (PMTE).

The WH, SST, CC, VTP and OTV variables were extracted daily and stratified by depth ranges (0–20 m, 20–50 m, 50–200 m), and the monthly average was calculated for all variables (for each stratum if necessary) and used in further data analysis.

### Data analysis

Daily abundance records of stranded seabirds were obtained from 257 beaches and aggregated into eight mesoregions. The abundance of stranding birds was summed for all beaches within each mesoregion and normalized by the total coastline distance (km) of the mesoregion. Temporal abundance was calculated seasonally, as seasonal trends are more influential on seabirds’ physiological patterns. Observations were structured by mesoregion and season. Stranding seabird responses were investigated with the abundance, richness, and diversity within each mesoregions and season combination. In this way we avoid the excess of zeros in our matrix and provide a very robust trend of temporal and spatial stranding variation within the Brazilian coastline. Species contributing fewer than 10 individuals across all observations (i.e., eight mesoregions over four years) were excluded, as they likely represent random stranding events with minimal impact on the overall pattern. This resulted in a dataset comprising 25 seabird species (Table 2).

**Table 2:** Stranding seabird species in the study. Description of stranding seabird species according to the status and number of individuals from 2016-2019. *  Near threatened, ** Vulnerable, ***Endangered (IUCN).

Status	Species	Abundance
Resident	*Fregata magnificens*	632
	*Larus dominicanus*	2862
	*Sterna hirundinacea*	107
	*Sula leucogaster*	2010
	*Thalasseus acuflavidus*	88
	*Thalasseus maximus*	18
Southern	*Ardenna gravis*	110
	*Ardenna grisea* *	77
	*Daption capense*	18
	*Fulmarus glacialoides*	10
	*Macronectes giganteus*	121
	*Oceanites oceanicus*	31
	*Pachyptila belcheri*	10
	*Pachyptila desolata*	14
	Procellaria aequinoctialis**	663
	Pterodroma incerta***	16
	*Pterodroma mollis*	13
	*Stercorarius chilensis*	12
	Thalassarche chlororhynchos***	979
	*Thalassarche melanophris* *	512
Northern	*Calonectris diomedea*	160
	*Calonectris borealis*	102
	*Puffinus puffinus*	3056
	*Stercorarius parasiticus*	12
	*Sterna hirundo*	67

Three separate models were developed to investigate spatial and temporal variation in stranded seabird abundance, richness, and diversity. Prior to modeling, data exploration accounted for influential observations, collinearity between covariates, residual heterogeneity, and autocorrelation. Residuals were analyzed using the Durbin-Watson test for autocorrelation and plotted to check for heteroskedasticity. Generalized least squares (GLS) regression addressed spatially correlated errors arising from observations collected at consecutive beaches. The best model was chosen with backwards elimination of non-significant covariates and goodness of fit was assessed through the lowest AIC (Akaike Information Criterion). Extreme stranding events (outliers) were observed in spring 2016, and we performed all analyses with and without these outliers to understand if and how this extremes event could affect our results. Separate models were also constructed for seabird migratory behavior: Resident, Southern Migrant and Northern Migrant, to determine whether overall patterns were influenced by behavior-specific factors.

Environmental and anthropogenic variables were combined in a single predictor matrix. Predictor variables collinearity was assessed by means of a Pearson correlation analysis and highly correlated variables (>0.8) were removed before modelling stranding bird’s responses (S1 Fig). The predict variables kept and used for modeling tests were: Sea surface temperature (ºC), concentration of chlorophyll (ug/L) (both used as a *proxy* for primary productive), wave height (m), wind intensity (m/s), wind direction, vessel traffic operated by Petrobras and other vessel traffic.

All statistical analyses were performed using the R software version 4.4.2 [31]. For more information about the models and approaches used here, please consult the Supporting Information (See S1 File).

## Results

Between January 2016 and December 2019, a total of 11759 stranded individuals were recorded along the Brazilian coast, representing 43 seabird species, eight families and three orders. After excluding rare stranding species, the analysis focused on 11,700 individuals belonging to 25 species. Of these, 64% were from the order Procellariiformes (n = 16), 28% Charadriiformes (n = 7) and 8% Suliformes (n = 2). We registered 6 resident species, totaling 5717 stranded individuals, 5 northern migratory species with 3397 individuals and 14 southern migratory species with 2586 individuals. Notably, 2247 individuals belonged to five species classified as threatened to some degree (Table 2, S2 Fig). Only 95 stranded birds (2016 =  37; 2017 =  16; 2018 =  11; 2019 =  31) from 13 species showed signs of oiling, with 68% (n = 63) of these being Manx shearwater (*Puffinus puffinus*).

To access the tables with the statistical results referenced throughout this section, please refer to the Supporting Information (See S1 File).

### Abundance of stranding seabirds

**Fig 2:**
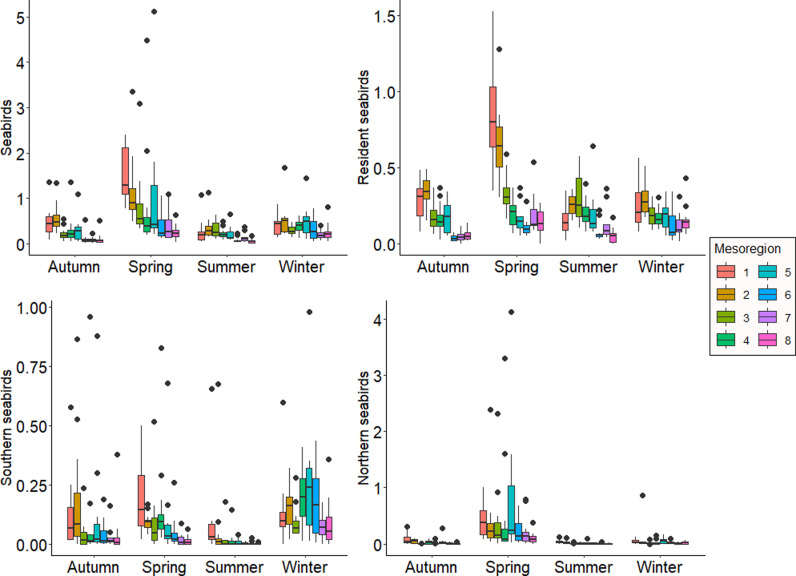
Abundance patterns of seabird strandings. Seabird stranding abundance patterns along the spatial and temporal gradients. Abundance corrected for sampling effort in km. a) all seabird species; b) resident seabird species; c) southern seabird species; d) northern seabird species.

**Fig 3:**
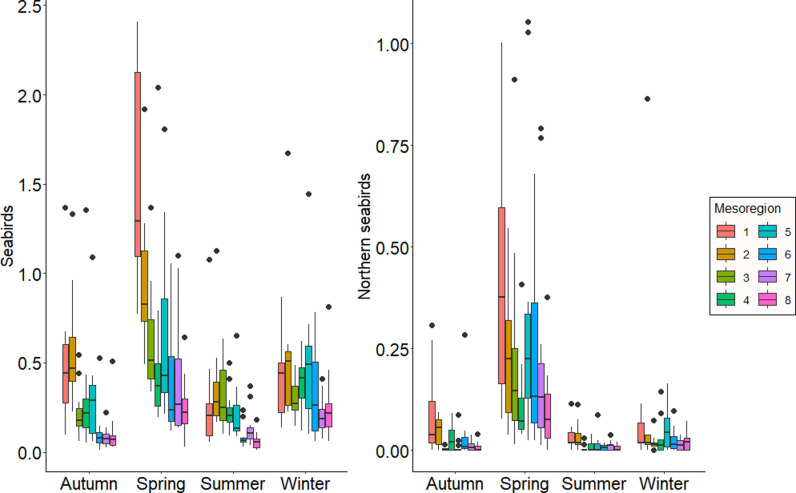
Abundance patterns of seabird strandings without outliers. Seabird stranding abundance patterns along the spatial and temporal gradients without extreme stranding events occurring in 2016. Abundance corrected for sampling effort in km. a) all seabird species; b) northern seabird species.

Between January and December 2019, the abundance of seabird strandings varied significantly across mesoregions and seasons (mesoregion: F-value =  22.78, p < 0.001; season: F-value = 23.50, p < 0.001). Significant differences were observed between most mesoregions (i.e.,: 1 x 8). Stranding abundance decreased from south to northern areas in all seasons, however, higher strand abundance was consistently detected in spring. In contrast, autumn and summer showed lower but similar trends, while winter exhibited no major spatial differences in seabird stranding abundances (Fig 2a). Nevertheless, winter recorded the second-highest number of stranding events. Similar trends were observed even after excluding extreme stranding events from spring 2016 (Fig 3a).

The abundance of resident seabird strandings along the coast depended on the season (mesoregion*season = F-value = 3.40, p < 0.001). Differences of South and Central Coast of Santa Catarina (mesoregions 1 and 2) and all other regions northward occurred during spring (Fig 2b). Among the six resident species, three showed higher stranding abundance, Kelp gulls (*Larus dominicanus*) (n = 2862), Bown booby (*Sula leucogaster*) (n = 2010) and Magnificent frigatebird (*Fregata magnificens*) (n = 632).

Migratory movements influenced stranding patterns. Southern species showed trends similar to those of all seabird species, with significant differences across mesoregions and seasons (mesoregion: F-value =  17.25, p < 0.001; season: F-value = 20.52, p < 0.001). Summer had the lowest abundance (n = 153), with no significant differences in autumn (n = 605) and spring (n = 630). Winter exhibited the highest stranding abundance (n = 1,198), particularly in central mesoregions (4, 5 and 6) (Fig 2c). The three southern species with the highest stranding records were Atlantic yellow-nosed (*Thalassarche chlororhynchos*) (n = 979), White-chinned petrel (*Procellaria aequinoctialis*) (n = 663) and Black-browed albatross (*Thalassarche mellanophris*) (n = 512), all of which are listed as threatened by the IUCN Red List of Threatened Species.

Northern species stranded less, and spatial differences were not related to seasonality (mesoregion: F value =  22.09; p < 0.001, season: F value =  109.11; p < 0.001). Higher stranding abundances occurred in the extreme south of Santa Catarina, with a decreasing trend toward the north coast of São Paulo. No significant differences were observed between mesoregion 5 and the first two. Spring had the highest stranding abundance of all seasons, totaling 2,816 stranded individuals. In 2016, six events accounted for 1,111 stranded individuals, with 1,107 of these being Manx Shearwater (*Puffinus puffinus*). Even after excluding these six events, the spatial and temporal stranding patterns remained consistent (Fig 3b).

When analyzing the influence of environmental variables on stranding abundance of all seabird species, results showed significantly higher stranding abundance with increasing wind intensity and wave height, and lower abundance when sea surface temperature was slightly higher (S3 Fig). Nonetheless, the effect size of these variables were estimated to be very small.

**Fig 4:**
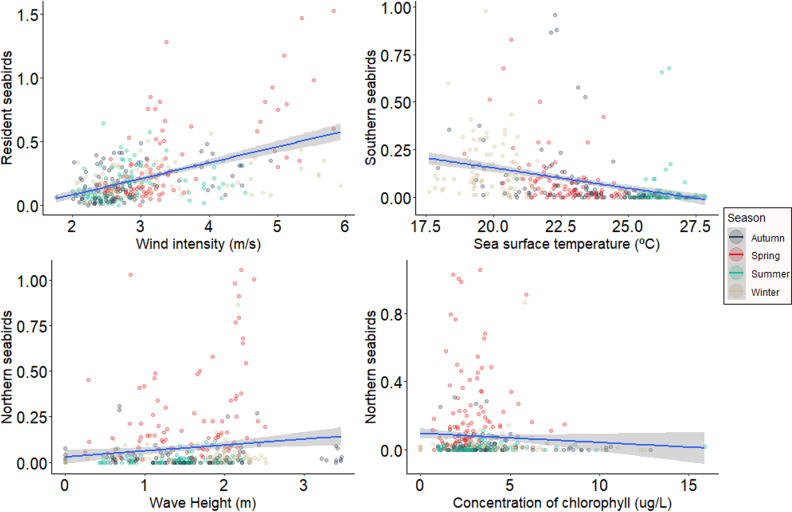
Response of stranding abundance according to status in relation to the environmental predictors. Scatter diagrams values between resident, southern and northern seabirds stranding abundance and environmental variables were significant value of the test (p).

Environmental variables had minimal effects on the stranding of resident and migratory species. Resident species exhibited higher stranding with increased wind intensity. Southern species showed higher stranding when sea surface temperature was lower, while northern species stranding was higher with increased wave height and chlorophyll concentration. This result held even after running the analysis without the extreme stranding events from spring 2016 (Fig 4).

### Stranding richness

Species richness varied significantly across mesoregions and seasons (mesoregion: F-value =  72.71, p < 0.001; season: F-value = 56.77, p < 0.001), with decreasing stranding richness from south to northern areas in all seasons (S4a Fig). Significant differences were observed between the most distant mesoregions (i.e., Mesoregion 1–8). Winter and spring presented a higher stranding richness, while summer presented the lowest.

Regardless of migration behavior, stranding richness decreased from south to northern mesoregions. Resident species stranding differed both spatially and temporally (mesoregion: F-value =  78.76, p < 0.001; season: F-value = 5.89, p < 0.001), with no large stranding events (outliers) (S4b Fig). The consistent spatial and temporal trends may indicate that the stranding of the six resident species may be linked to their spatial range.

Southern migratory species strandings were higher in south (14 spp.) and decreased toward northern areas (12 spp.) (mesoregion: F value =  39.57, p < 0.001; season: F value = 70.47, p < 0.001). Southern migratory species stranded more during winter and less during summer. (S4c Fig). Northern species (5 spp.) stranding was higher in the south and decreased toward northern areas (mesoregion: F value =  49.99, p < 0.001; season: F value = 16.31, p < 0.001). Higher strandings were detected during spring and winter (S4d Fig).

Stranding seabird richness was significantly higher with increasing wave height, wind intensity and chlorophyll concentration, and slightly lower with increasing sea surface temperature (S5 Fig). However, as observed for stranding abundance, the effect size of these variables was estimated to be small.

**Fig 5:**
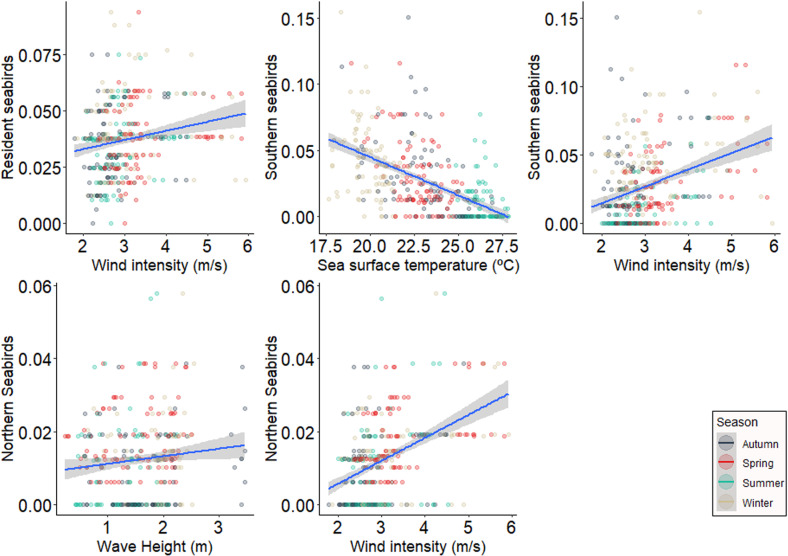
Response of stranding richness according to status in relation to the environmental predictors. Scatter diagrams values between resident, southern and northern seabird stranding richness and environmental variables were significant value of the test (p).

Resident species strandings were positively correlated with wind intensity. Southern species stranded more when sea surface temperature was lower and wind intensity was higher. Northern seabird species stranded more with higher wave height and wind intensity (Fig 5).

### Stranding diversity–Hills number (Shannon exp (H’))

As shown for the other indices, seabird species diversity was higher in the south and decreased toward northern areas, regardless of migratory behavior (mesoregion: F-value = 93.96, p < 0.001). Higher seabird diversity occurred during winter and was lowest during summer (season: F-value = 7.88, p < 0.001) (S6a Fig).

Resident seabird diversity spatial differences depending on the season (F-value = 4.14, p < 0.001), as higher stranding diversity was only detected during winter (t-value = 2.39, p = 0.018) (S6b Fig). Resident diversity stranding was higher in the south and lowest in the north, although no major differences were detected.

Southern species diversity decreased from south to northern areas, with lower stranding diversity during summer (mesoregion: F-value =  43.33, p < 0.001; season: F-value = 67.34, p < 0.001) (S6c Fig). Northern species stranding diversity decreased from south to northern areas, with higher strandings during summer and winter (mesoregion: F-value =  93.96, p < 0.001; season: F-value = 7.88, p < 0.001) (S6d Fig).

There was no strong effect of environmental or anthropogenic variables on seabird stranding diversity. Sea surface temperature presented a negative correlation with stranding diversity, while concentration of chlorophyll showed a positive correlation (S7 Fig).

**Fig 6:**
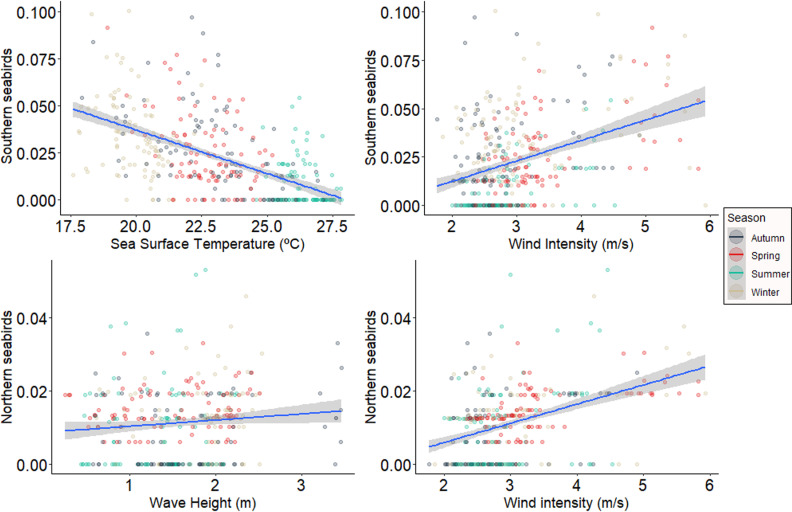
Response of stranding diversity according to status in relation to the environmental predictors. Scatter diagrams values between southern and northern seabird stranding diversity and environmental variables were significant value of the test (p).

The diversity of resident seabird strandings was not associated with any predictor variables. Southern migratory seabird diversity decreased with higher sea surface temperature and increased with wind intensity. Northern seabird species diversity was positively correlated with wave height and wind intensity (Fig 6).

## Discussion

Our study revealed a consistent downward trend in strandings from southern to northern mesoregions along the Brazilian coast for all ecological community indices, regardless of migratory behavior. We attribute this pattern to multifactorial causes, including ecological processes such as home range, breeding, and migratory behaviors of seabirds in the Santos Basin [17,32]. Additionally, environmental variables influence oceanographic processes [33], the drift of stranded birds [10,34] and climatic factors, such as storms, that can increase bird mortality [16]. By analyzing the results separately based on the geographic origin of the species (whether resident or migratory), we identified how different behaviors contribute to the overall community pattern and how environmental predictors influence our findings.

Notably, less than 0.8% of the stranded birds in this study showed signs of oiling, indicating that oil contamination did not influence the observed stranding patterns. Moreover, no significant relationship was found with vessel traffic intensity, contrary to our third hypothesis. However, incidents in the oil exploration and production chain in the Santos Basin may directly impact seabird species that use the area for nesting and foraging, given the region’s high sensitivity and environmental vulnerability [35,36]. Among the 95 oiled birds, 63 were Manx shearwater (*Puffinus puffinus*), a species classified as having extreme sensitivity to oil spills according to the Bird Sensitivity to Oil Index (BSOI) [37]. Additionally, of the 31 recorded incidents in 2019, 20 occurred between October and December, shortly after the environmental disaster in early September of the same year, during which crude oil reached the tropical beaches of northeastern Brazil. By the end of December, oil slicks had been recorded as far south as the state of Rio de Janeiro [38]. Of these incidents, 18 involved Manx shearwater, whose migration route aligns with the movement of the crude oil along the Brazilian coast [38,39]. This suggests that contamination may have occurred during migration. While oil contamination did not significantly influence the overall stranding patterns, the high sensitivity of species like the Manx shearwater to oil spills underscores their vulnerability. This finding highlights the need for ongoing monitoring and preventive measures, particularly given the environmental sensitivity of the Santos Basin and surrounding areas.

For resident species, a general south-to-north decline in strandings was observed, with slight temporal variations in ecological indices. Abundance peaked in spring, while species richness and diversity were highest in winter, partially supporting for our first hypothesis. The increased strandings in spring likely reflect reproductive peaks and fledglings [40]. Spatial variation aligns with expectations, as mesoregions 1 to 4 (Santa Catarina coast) host larger breeding colonies [32,41] compared to mesoregions 5 to 8, which encompass the Paraná and São Paulo coasts [42,43]. Similar patterns have been previously observed, with resident species strandings being concentrated near breeding colonies [34].

However, richness and diversity were higher during winter, particularly in mesoregions 2 and 3, suggesting that this pattern may be linked to the feeding behaviors and dietary preferences of resident species. Studies indicate that pelagic and demersal fish species like Sciaenidae, Engraulidae, and Clupeidae dominate seabird diets [44–49]. Many fish species in these families reach reproductive peaks during winter and spring, when lower temperatures favor spawning and larval survival, enhancing prey richness and diversity [50–53]. Limited dietary overlap [48] and diverse foraging strategies (such as dipping, surface plunging, parasitism, and plunge diving) [54], minimizes interspecific competition, boosting seabird richness and diversity. Besides these, artisanal shrimp fishing in Santa Catarina provides fishery discards that serve as a high-quality food source for many species, including Kelp gulls (*Larus domincanus*) [55], Magnificent frigatebird (*Fragata magnificens*) and terns (*Thalasseus acuflavidus, Sterna hirundinacea, Thalasseus maximus*) [56].

Wind intensity was positively correlated with increased abundance and richness of resident species strandings. Literature widely recognizes wind intensity as a key factor in driving the drift of stranded tetrapods to beaches [10,12,57,58]. Furthermore, environmental variables such as wind intensity influence fish assemblages, affecting fishery yields and food availability for seabirds [59,60]. This dynamic plays a critical role in both seabird mortality and carcass delivery to coastal areas [61].

Southern hemisphere species exhibited the lowest abundance of strandings among all groups. Their stranding patterns in mesoregions correspond closely to their migratory processes, supporting our first hypothesis. Higher abundance in the southernmost mesoregions aligns with findings from studies evaluating oceanic species interacting with fishing vessels, which observed greater abundance in sub-areas of Santa Catarina and Paraná, compared to Rio de Janeiro and São Paulo [43,62].

However, these species were the main contributors to winter being the season with the highest seabird richness and diversity. This is because they are regular non-breeding visitors from the south [24,27]. The atlantic yellow-nosed albatross (*Thalassarche chlororhynchos*), Black-browed albatross (*Thalassarche melanophris*), White-chinned petrel (*Procellaria aequinoctialis*), Sooty shearwater (*Ardenna grisea*), Southern giant petrel (*Macronectes giganteus*) and Great shearwater (*Ardenna gravis*) are species that commonly breed in the islands of Tristan da Cunha and Gough, Patagonia, South Georgia and/or Falkland Islands [13,24,41]. These species are commonly found along the southern and southeastern coast of Brazil, mainly foraging in these areas during winter and spring months [24,45]. This seasonal pattern is primarily driven by the Malvinas/Falklands Current [63], which influences the zonal variation of the Brazil-Malvinas confluence, altering sea surface temperature, and, consequently, altering primary productivity and pray availability [64].

During winter, the confluence region serves as a critical foraging area for many species, including mollymawks (*Thalassarche* spp.) and petrels (*Procellaria* spp., *Macronectes giganteus*) [65]. This is likely due to the massive presence of migratory cephalopods, such as *Illex argentinus*, originating from Argentine waters [66], which are a important dietary component for these birds [65,67,68]. These squid congregate along the continental slope and shelf break and represent the most important squid species harvested by fisheries in the southern Brazil [69,70]. Notably, *I. argentinus* migrates to the Brazilian coast during winter, particularly to deeper waters, where it finds suitable conditions for reproduction [70,71]. Similar to resident seabird species, the availability of trawl fishery discards facilitates dietary overlap and the coexistence of these non-breeding migratory seabirds, thereby enhancing both richness and diversity within the community [56,65]. The oceanographic patterns may explain why we found a negative correlation between sea surface temperature and strandings in all ecological indices for southern species, where lower temperatures (~17ºC) were associated with higher stranding events. This finding contradicts our expectations in the second hypothesis and suggests that environmental variables may correlate differently depending on the species being evaluated. For resident seabirds, higher wind intensity was associated with higher richness and diversity of stranded birds, highlighting the importance of considering this predictor in studies aimed to understanding marine fauna strandings.

Five species of stranded seabirds identified in this study belong to the order Procellariiformes from the Southern Hemisphere, an order with high percentage of threatened species [72,73]. Populations of large albatrosses and petrels have significantly declined in recent years [74], due to various factors, including invasive alien species at breeding sites, pollution (light and plastic ingestion), bycatch, and overfishing [73–76]. The last three factors may also be directly to the stranding process. Birds weakened by long migratory journeys and facing these threats are often driven by winds, where they strand.

We observed that the abundance of northern seabird strandings was higher during spring, supporting our first hypothesis. However, this pattern differed spatially from the community, primarily due to six mass stranding events in spring 2016, predominantly involving Manx Shearwater. This species is a remarkable transequatorial migrant [24], wintering off the Patagonian Shelf in the western South Atlantic [39], and along the Brazilian coast, mainly during austral spring (September to December) [77]. Studies on this species along the Brazilian coast have shown significant spatial variability, with records from the state of Rio Grande do Sul [78,79], São Paulo [80], Rio de Janeiro and Espírito Santo [77], peaking between September and December [81]. The mortality of Manx shearwaters was linked to indirect effects of large-scale climate conditions, particularly the 2015–2016 El Niño event, which caused environmental disruptions such as changes in prey communities and a decline in Brazilian sardines [16,82–84]. In this study, we observed that wave weight was positively correlated with increased stranding abundance. Wave height, a known proxy for storm intensity [16], may help explain the high stranding event of Manx shearwaters in 2016.

Nonetheless, when excluding the six mass stranding events, the abundance pattern along the coast remained consistent across other migratory behaviors and for the overall community. Like Manx shearwaters, other northern hemisphere species (Cory’s shearwaters, Common terns and Arctic skua) also undertake long transoceanic migrations to wintering areas in the South Atlantic [85]. Several stopover areas along the Brazilian coast used by these species [39] are primarily associated with high productivity waters [86]. The tendency of these birds to seek high-productivity areas may explain our findings, where chlorophyll concentration (proxy for productivity) was negatively correlated with stranding abundance. In areas of lower productivity and prey availability, these birds may be more susceptible to mortality, likely due to starvation [87]. Richness and diversity of these species were positively correlated with wind intensity and wave height, which were the most significant predictors of mortality for migratory species on the Brazilian coast [16].

## Conclusions

We observed a clear decreasing trend in seabird strandings across all ecological indices for the Santos Basin community, with the highest number of stranding events occurring in the southern mesoregions and progressively decreasing along the coast towards the northern mesoregions. The subtle variations observed in the indices across different migratory behaviors support our hypothesis that the pattern is primarily linked to the reproductive peaks of resident species and well-defined migratory periods for migratory birds, regardless of their hemisphere of origin, combined with their key dietary components. In addition, our findings identified environmental variables as robust predictors of seabirds strandings, including higher wave height and wind intensity, elevated chlorophyll concentration, and lower sea surface temperature. However, we suggest that these variables may correlate differently depending on the species evaluated. Despite the low number of oiled animals observed, we emphasize that seabirds, particularly migratory species, are highly vulnerable to oil spill contamination. This highlights the importance of monitoring projects such as the one developed by the PMP-BS, which plays a crucial role in understanding and mitigating the impacts of oil pollution on these vulnerable species. This study provides crucial insights into the dynamics of seabird species in the Santos Basin and the stranding processes associated with environmental factors. The findings contribute valuable data for conservation efforts, which could support development of effective management strategies and the establishment of new protected areas in the Santos Basin.

## Supporting Information

S1 Fig Pearson correlation matrix with all environmental and anthropogenic variables. (TIF)

S2 Fig The 25 seabird that contributed with more than 10 individuals to the role data set and were used for all analyses. (TIF)

S3 Fig Response of stranding abundance to the environmental predictors. Scatter diagrams values between all seabird stranding abundance and environmental variables were significant value of the test (p).
(TIF)

S4 Fig Richness patterns of seabird strandings. Stranding species richness patterns along the spatial and temporal gradients. Species richness corrected for sampling effort in km. a) all seabird species; b) resident seabird species; c) southern seabird species; d) northern seabird species.
(TIF)

S5 Fig Response of stranding richness to the environmental predictors. Scatter diagrams values between all seabird stranding richness and environmental variables were significant value of the test (p).
(TIF)

S6 Fig Diversity patterns of seabird strandings. Stranding species diversity patterns along the spatial and temporal gradients. Species richness corrected for sampling effort in km. a) all seabird species; b) resident seabird species; c) southern seabird species; d) northern seabird species.
(TIF)

S7 Fig Response of stranding diversity to the environmental predictors. Scatter diagrams values between all seabird stranding diversity and environmental variables were significant value of the test (p).
(TIF)

S1 File Supporting information. (DOCX)
