# Supplementary figures and images for: Seabird strandings on the Brazilian coast: What influences spatial and temporal patterns?
Source: PLoS One. 2025 Apr 16;20(4):e0317335. doi: 10.1371/journal.pone.0317335 (PMC12002494; doi:10.1371/journal.pone.0317335)

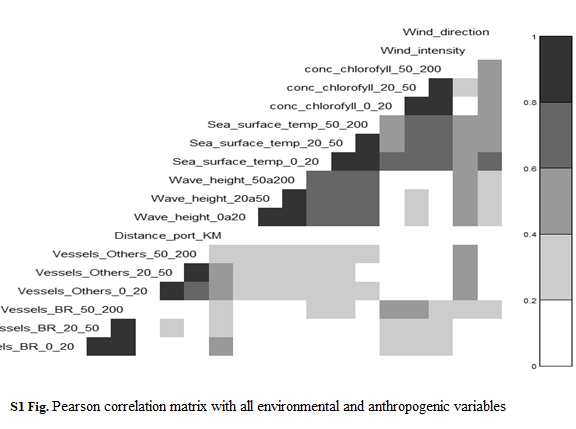

Supplement: S1 Fig — (TIF) [file pone.0317335.s001.tif]

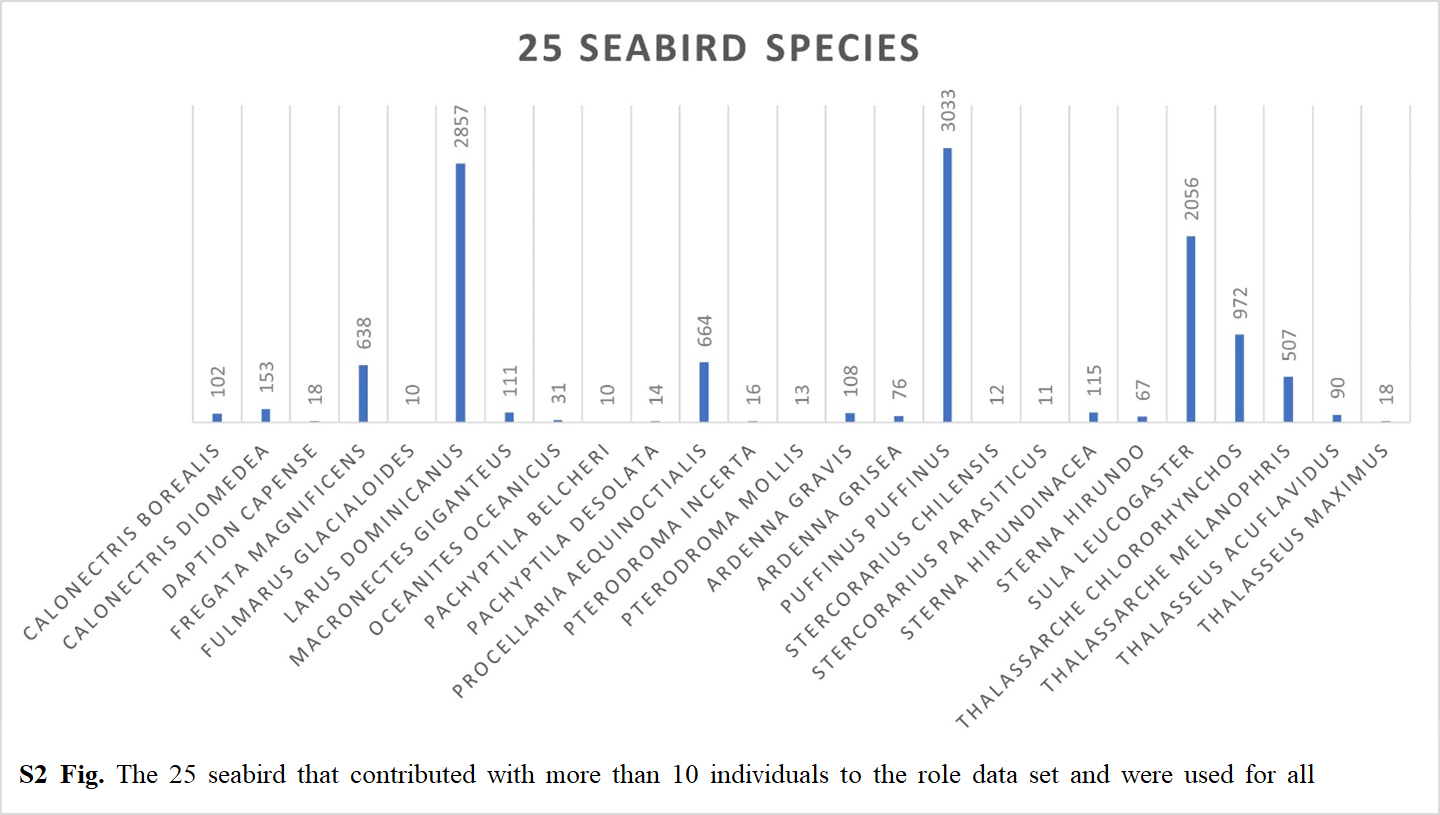

Supplement: S2 Fig — (TIF) [file pone.0317335.s002.tif]

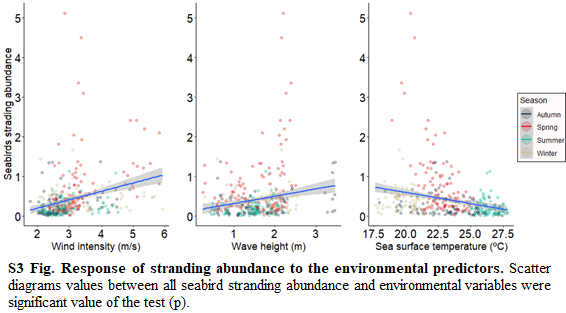

Supplement: S3 Fig — Scatter diagrams values between all seabird stranding abundance and environmental variables were significant value of the test (p). (TIF) [file pone.0317335.s003.tif]

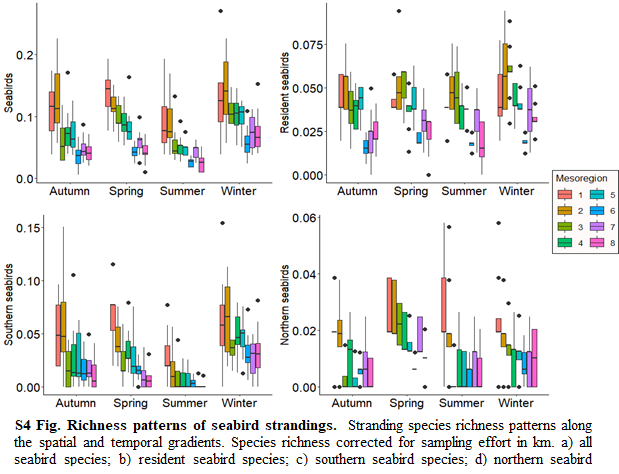

Supplement: S4 Fig — Stranding species richness patterns along the spatial and temporal gradients. Species richness corrected for sampling effort in km. a) all seabird species; b) resident seabird species; c) southern seabird species; d) northern seabird species. (TIF) [file pone.0317335.s004.tif]

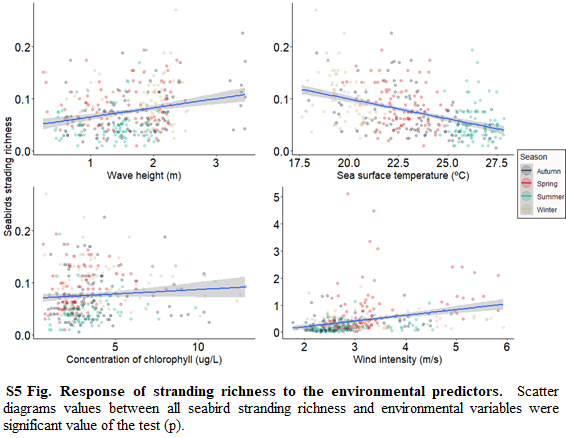

Supplement: S5 Fig — Scatter diagrams values between all seabird stranding richness and environmental variables were significant value of the test (p). (TIF) [file pone.0317335.s005.tif]

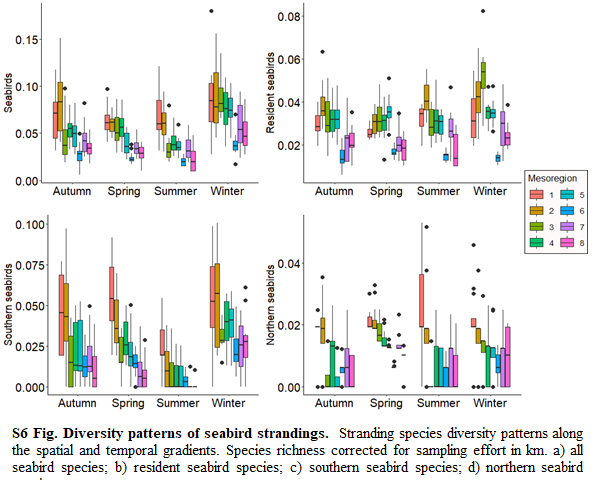

Supplement: S6 Fig — Stranding species diversity patterns along the spatial and temporal gradients. Species richness corrected for sampling effort in km. a) all seabird species; b) resident seabird species; c) southern seabird species; d) northern seabird species. (TIF) [file pone.0317335.s006.tif]

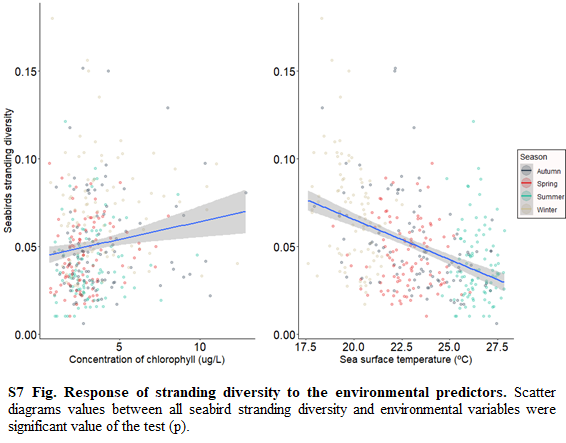

Supplement: S7 Fig — Scatter diagrams values between all seabird stranding diversity and environmental variables were significant value of the test (p). (TIF) [file pone.0317335.s007.tif]
