# Supplementary material for: Seabird strandings on the Brazilian coast: What influences spatial and temporal patterns?
Source: PLoS One. 2025 Apr 16;20(4):e0317335. doi: 10.1371/journal.pone.0317335 (PMC12002494; doi:10.1371/journal.pone.0317335)
Supplement: S1 File — (DOCX) [file pone.0317335.s008.docx]

**Supporting information**

*Species Data*

The records of stranding birds for 8 mesoregions in four years of study included 74 species, of which 44 were considered seabirds, 9 shorebirds and 16 waterbirds. We only used for this study seabird species that contributed with more 10 individuals to the role data set.

**S1 Table - The total bird species that stranded in the study area between 2016 and 2019 years**.

| **Specie** | **Stranding Events** | **English name** | **Behavior** | **Status**^a,b,c^ | **Status II**^a^ | **IUCN** | **Population**^b^ |
| --- | --- | --- | --- | --- | --- | --- | --- |
| *Anous stolidus* | 8 | Brown Noddy | Seabird | Resident | BR | Least Concern | Stable |
| *Ardea alba* | 39 | Great White Egret | Waterbirds | Resident | BR | Least Concern | Unknown |
| *Ardea cocoi* | 16 | Cocoi Heron | Waterbirds | Resident | BR | Least Concern | Increasing |
| *Botaurus pinnatus* | 2 | Pinnated Bittern | Waterbirds | Resident | BR | Least Concern | Unknown |
| *Bubulcus ibis* | 3 | Cattle Egret | Waterbirds | Resident | BR | Least Concern | Increasing |
| *Butorides striata* | 6 | Striated Heron | Waterbirds | Resident | BR | Least Concern | Decreasing |
| *Calidris alba* | 10 | Sanderling | Shorebird | Northern Migratory | VI (N) | Least Concern | Unknown |
| *Calidris canutus* | 4 | Red Knot | Shorebird | Northern Migratory | VI (N) | Near Threatened | Decreasing |
| *Calidris fuscicollis* | 7 | White-rumped Sandpiper | Shorebird | Northern Migratory | VI (N) | Least Concern | Decreasing |
| *Calonectris borealis* | 102 | Cory's Shearwater | Seabird | Northern Migratory | VI (E) | Least Concern | Unknown |
| *Calonectris diomedea* | 153 | Scopoli's Shearwater | Seabird | Northern Migratory | VA (E) | Least Concern | Decreasing |
| *Calonectris edwardsii* | 1 | Cape Verde Shearwater | Seabird | Northern Migratory | VI (E) | Near Threatened | Decreasing |
| *Charadrius collaris* | 2 | Collared Plover | Waterbirds | Resident | BR | Least Concern | Decreasing |
| *Charadrius semipalmatus* | 7 | Semipalmated Plover | Waterbirds | Northern Migratory | VI (N) | Least Concern | Stable |
| *Chloroceryle americana* | 1 | Green Kingfisher | Waterbirds | Resident | BR | Least Concern | Decreasing |
| *Chroicocephalus cirrocephalus* | 1 | Gray-hooded Gull | Seabird | Resident | BR | Least Concern | Stable |
| *Chroicocephalus maculipennis* | 3 | Brown-hooded Gull | Seabird | Resident | BR | Least Concern | Increasing |
| *Daption capense* | 18 | Cape Petrel | Seabird | Southern Migratory | VI (S) | Least Concern | Stable |
| *Diomedea epomophora* | 2 | Royal Albatross | Seabird | Southern Migratory | VI^#^ | Vulnerable | Stable |
| *Egretta caerulea* | 3 | Little Blue Heron | Waterbirds | Resident | BR | Least Concern | Decreasing |
| *Egretta thula* | 35 | Snowy Egret | Waterbirds | Resident | BR | Least Concern | Increasing |
| *Fregata magnificens* | 638 | Magnificent Frigatebird | Seabird | Resident | BR | Least Concern | Decreasing |
| *Fulmarus glacialoides* | 10 | Southern Fulmar | Seabird | Southern Migratory | VI (S) | Least Concern | Stable |
| *Gallinago paraguaiae* | 1 | South American Snipe | Waterbirds | Resident | BR | Least Concern | Stable |
| *Haematopus palliatus* | 22 | American Oystercatcher | Shorebird | Resident | BR | least Concern | Stable |
| *Halobaena caerulea* | 1 | Blue Petrel | Seabird | Southern Migratory | VA (S) | least Concern | Decreasing |
| *Himantopus melanurus* | 23 | White-backed Stilt | Shorebird | Resident | BR | Least Concern | Unknown |
| *Ixobrychus exilis* | 2 | Least Bittern | Waterbirds | Resident | BR | Least Concern | Stable |
| *Larus atlanticus* | 1 | Olrog's Gull | Seabird | southern Migratory | VI (S) | Near Threatened | Stable |
| *Larus dominicanus* | 2857 | Kelp Gull | Seabird | Resident | BR | Least Concern | Increasing |
| *Macronectes giganteus* | 111 | Southern Giant-Petrel | Seabird | Southern Migratory | VI (S) | Least Concern | Increasing |
| *Macronectes halli* | 5 | Northern Giant-Petrel | Seabird | Southern Migratory | VI (S) | Least Concern | Increasing |
| *Megaceryle torquata* | 1 | Ringed Kingfisher | Waterbirds | Resident | BR | least Concern | Stable |
| *Nyctanassa violácea* | 5 | Yellow-crowned Night-Heron | Waterbirds | Resident | BR | least Concern | Stable |
| *Nycticorax nycticorax* | 28 | Black-crowned Night-Heron | Waterbirds | Resident | BR | least Concern | Decreasing |
| *Oceanites oceanicus* | 31 | Wilson's Storm-Petrel | Seabird | Southern Migratory | VI (S) | Least Concern | Stable |
| *Pachyptila belcheri* | 10 | Slender-billed Prion | Seabird | Southern Migratory | VI (S) | Least Concern | Stable |
| *Pachyptila desolata* | 14 | Antarctic Prion | Seabird | Southern Migratory | VI (S) | Least Concern | Decreasing |
| *Pachyptila vittata* | 2 | Broad-billed Prion | Seabird | Southern Migratory | VA (S) | Least Concern | Decreasing |
| *Nannopterum brasilianum* | 926 | Neotropic Cormorant | Waterbirds | Resident | BR | Least Concern | Increasing |
| *Phimosus infuscatus* | 5 | Bare-faced Ibis | Waterbirds | Resident | BR | Least Concern | Stable |
| *Pluvialis dominica* | 5 | American Golden-Plover | Shorebird | Northern Migratory | VI (N) | Least Concern | Decreasing |
| *Pluvialis squatarola* | 1 | Black-bellied Plover | Shorebird | Northern Migratory | VI (N) | Least Concern | Decreasing |
| *Porphyrio Martinica* | 1 | Purple Gallinule | Waterbirds | Resident | BR | Least Concern | Decreasing |
| *Procellaria aequinoctialis* | 664 | White-chinned Petrel | Seabird | Southern Migratory | VI (S) | Vulnerable | Decreasing |
| *Procellaria conspicillata* | 3 | Spectacled Petrel | Seabird | Southern Migratory | VI (S) | Vulnerable | Increasing |
| *Pterodroma arminjoniana* | 1 | Trindade Petrel | Seabird | Resident | BR | Vulnerable | Stable |
| *Pterodroma incerta* | 16 | Atlantic Petrel | Seabird | Southern Migratory | VI (S) | Endangered | Decreasing |
| *Pterodroma mollis* | 13 | Soft-plumaged Petrel | Seabird | Southern Migratory | VI (S) | Least Concern | Stable |
| *Ardenna gravis* | 108 | Great Shearwater | Seabird | Southern Migratory | VI (S) | Least Concern | Stable |
| *Ardenna grisea* | 76 | Sooty Shearwater | Seabird | Southern Migratory | VI (S) | Near Threatened | Decreasing |
| *Puffinus puffinus* | 3033 | Manx Shearwater | Seabird | Northern Migratory | VI (E) | least Concern | Unknown |
| *Rynchops niger* | 39 | Black Skimmer | Waterbirds | Resident | BR | Least Concern | Decreasing |
| *Spheniscus magellanicus* | 18433 | Magellanic Penguin | Seabird | Southern Migratory | VI (S) | Least Concern | Decreasing |
| *Stercorarius antarcticus* | 4 | Brown Skua | Seabird | Southern Migratory | VI (S) | Least Concern | Decreasing |
| *Stercorarius chilensis* | 12 | Chilean Skua | Seabird | Southern Migratory | VI (S) | Least Concern | Stable |
| *Stercorarius longicaudus* | 4 | Long-tailed Jaeger | Seabird | Northern Migratory | VI (N, E?) | Least Concern | Stable |
| *Stercorarius maccormicki* | 6 | South Polar Skua | Seabird | Southern Migratory | VI (S) | Least Concern | Stable |
| *Stercorarius parasiticus* | 11 | Parasitic Jaeger | Seabird | Northern Migratory | VI (E, N?) | Least Concern | Stable |
| *Stercorarius pomarinus* | 6 | Pomarine Jaeger | Seabird | Northern Migratory | VI (E?, N?) | Least Concern | Stable |
| *Sterna hirundinacea* | 115 | South American Tern | Seabird | Resident | BR | Least Concern | Decreasing |
| *Sterna hirundo* | 67 | Common Tern | Seabird | Northern Migratory | VI (N, E) | Least Concern | Unknown |
| *Sterna trudeaui* | 7 | Snowy-crowned Tern | Seabird | Resident | BR | Least Concern | Stable |
| *Sula dactylatra* | 1 | Masked Booby | Seabird | Resident | BR | Least Concern | Decreasing |
| *Sula leucogaster* | 2056 | Brown Booby | Seabird | Resident | BR | Least Concern | Decreasing |
| *Syrigma sibilatrix* | 2 | Whistling Heron | Waterbirds | Resident | BR | Least Concern | Unknown |
| *Thalassarche chlororhynchos* | 972 | Yellow-nosed Albatross | Seabird | Southern Migratory | VI (S) | Endangered | Decreasing |
| *Thalassarche melanophris* | 507 | Black-browed Albatross | Seabird | Southern Migratory | VI (S) | Near Threatened | Decreasing |
| *Thalasseus acuflavidus* | 90 | Cabot's Tern | Seabird | Resident | BR, VI (S, N) | Least Concern | Unknown |
| *Thalasseus maximus* | 18 | Royal Tern | Seabird | Resident | BR, VI (S, N?) | Least Concern | Stable |
| *Tringa melanoleuca* | 3 | Greater Yellowlegs | Shorebird | Northern Migratory | VI (N) | Least Concern | Stable |
| *Tringa solitária* | 1 | Solitary Sandpiper | Shorebird | Northern Migratory | VI (N) | Least Concern | Decreasing |
| *Vanellus chilensis* | 3 | Southern Lapwing | Waterbirds | Resident | BR | Least Concern | Increasing |
| *Xema sabini* | 1 | Sabine's Gull | Seabird | Northern Migratory | VA (N?) | Least Concern | Stable |

^a^ [1]

^b^ [2]

^c^ [3]

*Data Analyses*

We constructed different models to understand whether the observed patterns for abundance, richness and diversity of stranded seabirds along the coast of Brazil were explained by the mesoregion and/or season in which they were found, and whether these patterns were influenced by migratory movements of seabirds and this was affected by anthropogenic and environmental variables. All models were conducted with Generalized Least Squares (GLS), due the heteroskedastic variance structure for the interaction between Mesoregion and Season, and we had a correlation between the residuals due to the spatial autocorrelation observed in the Mesoregion. We used in all models *varIdent* (form=~1|Mesoregion*Season) for corrected variance structure and *corCAR1* (form=~1|Mesoregion) for the spatial autocorrelation in Mesoregion.

- *Abundance*

First model with abundance of all seabirds species and Mesoregion and Season (GLS, abundance ~ Mesoregion + Season) showed a significant difference of mesoregion and season (mesoregion: p<0.001, F-value = 17.6991; season: p<0.0001, F-value= 18.3066).

**S2 Table. GLS for stranding abundance.** Results of generalized least squares (GLS) regression model of seabird stranding abundance spatial and temporal differences along the Brazilian coast. All variables with statistical significance are marked with an asterisk. (Reference level: mesoregion 1 in autumn)

| Parameter | Value | Std.Error | t-value | p-value |
| --- | --- | --- | --- | --- |
| (Intercept) | 0.360 | 0.061 | 5.872 | 0.000 |
| Mesoregion2 | 0.109 | 0.089 | 1.231 | 0.219 |
| Mesoregion3 | -0.147 | 0.065 | -2.247 | 0.025 |
| Mesoregion4 | -0.096 | 0.067 | -1.428 | 0.154 |
| Mesoregion5 | -0.098 | 0.077 | -1.261 | 0.208 |
| Mesoregion6 | -0.258 | 0.064 | -4.046 | 0.000 |
| Mesoregion7 | -0.264 | 0.062 | -4.261 | 0.000 |
| Mesoregion8 | -0.289 | 0.062 | -4.628 | 0.000 |
| Spring | 0.262 | 0.046 | 5.653 | 0.000 |
| Summer | 0.000 | 0.022 | -0.005 | 0.996 |
| Winter | 0.107 | 0.024 | 4.433 | 0.000 |

Residual standard error: 0.2948378

Parameter estimate Phi = 0.2415003

AIC = 47.1865; BIC= 221.0148; logLik= 20.40675

Similarly, three other models were conducted with the response variable been (1) Abundance of Resident seabirds, (2) Abundance of Southern seabirds, (3) Abundance of Northern seabirds, in order to understand the effects of this different behaviours on the overall seabird strandings.

1. Abundance of Resident seabirds (GLS, Resident abundance ~ Mesoregion * Season) showed a significant difference of mesoregion and season for all ecological indices (mesoregion: p<0.001, F-value = 44.8829; season: p<0.0001, F-value= 15.7659; mesoregion*season= p<0.001, F-value= 3.4000).

**S3 Table. GLS for resident seabird stranding abundance.** Results of generalized least squares (GLS) regression model of resident seabird stranding abundance spatial and temporal differences along the Brazilian coast. Only variables with statistical significance were held here. (Reference level: mesoregion 1*autumn)

| Parameter | Value | Std.Error | t-value | p-value |
| --- | --- | --- | --- | --- |
| Mesoregion3 | -0.118 | 0.051 | -2.308 | 0.022 |
| Mesoregion4 | -0.133 | 0.053 | -2.487 | 0.013 |
| Mesoregion5 | -0.116 | 0.054 | -2.132 | 0.034 |
| Mesoregion6 | -0.250 | 0.041 | -6.150 | 0.000 |
| Mesoregion7 | -0.240 | 0.042 | -5.769 | 0.000 |
| Mesoregion8 | -0.225 | 0.042 | -5.368 | 0.000 |
| Spring | 0.503 | 0.139 | 3.614 | 0.000 |
| Summer | -0.126 | 0.050 | -2.515 | 0.012 |
| Mesoregion3: Spring | -0.336 | 0.147 | -2.283 | 0.023 |
| Mesoregion4: Spring | -0.463 | 0.146 | -3.166 | 0.002 |
| Mesoregion5: Spring | -0.511 | 0.147 | -3.470 | 0.001 |
| Mesoregion6: Spring | -0.432 | 0.141 | -3.071 | 0.002 |
| Mesoregion7: Spring | -0.369 | 0.145 | -2.546 | 0.011 |
| Mesoregion8: Spring | -0.426 | 0.142 | -3.004 | 0.003 |
| Mesoregion3: Summer | 0.258 | 0.078 | 3.321 | 0.001 |
| Mesoregion4: Summer | 0.189 | 0.067 | 2.832 | 0.005 |
| Mesoregion6: Summer | 0.171 | 0.054 | 3.137 | 0.002 |
| Mesoregion7: Summer | 0.210 | 0.062 | 3.404 | 0.001 |
| Mesoregion8: Summer | 0.127 | 0.054 | 2.361 | 0.019 |

Residual standard error: 0.1012566

Parameter estimate Phi = 0.2191884

AIC = -663.2792; BIC= -406.4874; logLik= 396.6369

1. Abundance of Southern seabirds (GLS, Southern abundance ~ Mesoregion + Season) showed a significant difference of mesoregion and season for all ecological indices (mesoregion: p<0.001, F-value = 17.24911; season: p<0.0001, F-value= 20.51772).

**S4 Table. GLS for southern seabird stranding abundance.** Results of generalized least squares (GLS) regression model of southern seabird stranding abundance spatial and temporal differences along the Brazilian coast. Only variables with statistical significance were held here. (Reference level: mesoregion 1and autumn)

| Parameter | Value | Std.Error | t-value | p-value |
| --- | --- | --- | --- | --- |
| (Intercept) | 0.137 | 0.031 | 4.457 | 0.000 |
| Mesoregion3 | -0.089 | 0.032 | -2.781 | 0.006 |
| Mesoregion4 | -0.078 | 0.033 | -2.377 | 0.018 |
| Mesoregion5 | -0.098 | 0.030 | -3.238 | 0.001 |
| Mesoregion6 | -0.104 | 0.030 | -3.438 | 0.001 |
| Mesoregion7 | -0.105 | 0.030 | -3.466 | 0.001 |
| Mesoregion8 | -0.107 | 0.030 | -3.550 | 0.000 |
| Summer | -0.029 | 0.010 | -3.017 | 0.003 |
| Winter | 0.048 | 0.013 | 3.553 | 0.000 |

Residual standard error: 0.1792142

Parameter estimate Phi = 0.1852204

AIC = -863.096; BIC= -689.2677; logLik= 475.548

1. Abundance of Northern seabirds (GLS, Northern abundance ~ Mesoregion + Season) showed a significant difference of mesoregion and season for all ecological indices (mesoregion: p<0.001, F-value = 22.0855; season: p<0.0001, F-value= 109.1065).

**S5 Table. GLS for northern seabird stranding abundance.** Results of generalized least squares (GLS) regression model of northern seabird stranding abundance spatial and temporal differences along the Brazilian coast. Only variables with statistical significance were held here. (Reference level: mesoregion 1and autumn)

| Parameter | Value | Std.Error | t-value | p-value |
| --- | --- | --- | --- | --- |
| (Intercept) | 0.117 | 0.009 | 12.933 | 0.000 |
| Mesoregion3 | -0.041 | 0.009 | -4.382 | 0.000 |
| Mesoregion4 | -0.038 | 0.011 | -3.615 | 0.000 |
| Mesoregion5 | -0.039 | 0.010 | -3.780 | 0.000 |
| Mesoregion6 | -0.062 | 0.009 | -6.820 | 0.000 |
| Mesoregion7 | -0.045 | 0.009 | -4.802 | 0.000 |
| Mesoregion8 | -0.054 | 0.009 | -5.981 | 0.000 |
| Spring | 0.207 | 0.029 | 7.258 | 0.000 |
| Summer | -0.036 | 0.004 | -10.280 | 0.000 |
| Winter | 0.040 | 0.005 | 8.102 | 0.000 |

Residual standard error: 0.02643496

Parameter estimate Phi = 0.2928212

AIC = -1012.024; BIC= -838.1958; logLik= 550.012

- *Richness*

First model with richness of all seabirds species and Mesoregion and Season (GLS, richness ~ Mesoregion + Season) showed a significant difference of mesoregion and season for all ecological indices (mesoregion: p<0.001. F-value = 72.7059; season: p<0.0001. F-value= 56.7698).

**S6 Table. GLS for seabird stranding richness.** Results of generalized least squares (GLS) regression model of seabird stranding richness spatial and temporal differences along the Brazilian coast. All variables with statistical significance are marked with an asterisk. (Reference level: mesoregion 1 in autumn)

| Parameter | Value | Std.Error | t-value | p-value |
| --- | --- | --- | --- | --- |
| (Intercept) | 0,114 | 0,007 | 15,809 | 0,000 |
| Mesoregion3 | -0,037 | 0,008 | -4,578 | 0,000 |
| Mesoregion4 | -0,043 | 0,008 | -5,461 | 0,000 |
| Mesoregion5 | -0,047 | 0,008 | -6,148 | 0,000 |
| Mesoregion6 | -0,077 | 0,007 | -10,837 | 0,000 |
| Mesoregion7 | -0,062 | 0,007 | -8,349 | 0,000 |
| Mesoregion8 | -0,076 | 0,007 | -10,254 | 0,000 |
| Spring | 0,010 | 0,003 | 2,994 | 0,003 |
| Summer | -0,013 | 0,003 | -4,153 | 0,000 |
| Winter | 0,028 | 0,004 | 7,064 | 0,000 |

Residual standard error: 0.04426471

Parameter estimate Phi = 0.07452281

AIC = -1673.078; BIC= -1499.25; logLik= 880.5391

Similarly, three other models were conducted with the response variable been (1) Richness of Resident seabirds, (2) Richness of Southern seabirds, (3) Richness of Northern seabirds, in order to understand the effects of this different behaviours on the overall seabird strandings.

1. Richness of Resident seabirds (GLS, Resident richness ~ Mesoregion + Season) showed a significant difference of mesoregion and season for all ecological indices (mesoregion: p<0.001, F-value = 78.751; season: p<0.0001, F-value= 5.891).

**S7 Table. GLS for resident seabird stranding richness.** Results of generalized least squares (GLS) regression model of resident seabird stranding richness spatial and temporal differences along the Brazilian coast. Only variables with statistical significance were held here. (Reference level: mesoregion 1*autumn)

| Parameter | Value | Std.Error | t-value | p-value |
| --- | --- | --- | --- | --- |
| (Intercept) | 0.040 | 0.002 | 18.266 | 0.000 |
| Mesoregion2 | 0.011 | 0.003 | 3.277 | 0.001 |
| Mesoregion3 | 0.008 | 0.003 | 2.606 | 0.010 |
| Mesoregion6 | -0.023 | 0.002 | -11.427 | 0.000 |
| Mesoregion7 | -0.011 | 0.003 | -3.815 | 0.000 |
| Mesoregion8 | -0.016 | 0.003 | -6.344 | 0.000 |
| Spring | 0.003 | 0.001 | 2.573 | 0.011 |
| Winter | 0.004 | 0.001 | 3.121 | 0.002 |

Residual standard error: 0.01140372

Parameter estimate Phi = 0.1589576

AIC = -2369.382; BIC= -2195.554; logLik= 1228.691

1. Richness of Southern seabirds (GLS, Southern richness ~ Mesoregion + Season) showed a significant difference of mesoregion and season for all ecological indices (mesoregion: p<0.001, F-value = 39.56680; season: p<0.0001, F-value= 70.47196).

**S8 Table. GLS for southern seabird stranding richness.** Results of generalized least squares (GLS) regression model of southern seabird stranding richness spatial and temporal differences along the Brazilian coast. Only variables with statistical significance were held here. (Reference level: mesoregion 1and autumn)

| Parameter | Value | Std.Error | t-value | p-value |
| --- | --- | --- | --- | --- |
| (Intercept) | 0.049 | 0.005 | 9.570 | 0.000 |
| Mesoregion3 | -0.029 | 0.005 | -5.510 | 0.000 |
| Mesoregion4 | -0.024 | 0.006 | -4.259 | 0.000 |
| Mesoregion5 | -0.025 | 0.005 | -4.637 | 0.000 |
| Mesoregion6 | -0.032 | 0.005 | -6.528 | 0.000 |
| Mesoregion7 | -0.034 | 0.005 | -6.738 | 0.000 |
| Mesoregion8 | -0.035 | 0.005 | -7.107 | 0.000 |
| Summer | -0.013 | 0.002 | -5.767 | 0.000 |
| Winter | 0.017 | 0.003 | 5.796 | 0.000 |

Residual standard error 0.02319488

Parameter estimate Phi = 0.1025074

AIC = -1999.087; BIC= -1825.259; logLik= 1043.544

1. Richness of Northern seabirds (GLS, Northern richness ~ Mesoregion + Season) showed a significant difference of mesoregion and season for all ecological indices (mesoregion: p<0.001, F-value = 49.9883; season: p<0.0001, F-value= 16.3058).

**S9 Table. GLS for northern seabird stranding richness.** Results of generalized least squares (GLS) regression model of northern seabird stranding richness spatial and temporal differences along the Brazilian coast. Only variables with statistical significance were held here. (Reference level: mesoregion 1and autumn)

| Parameter | Value | Std.Error | t-value | p-value |
| --- | --- | --- | --- | --- |
| (Intercept) | 0.020 | 0.002 | 8.976 | 0.000 |
| Mesoregion3 | -0.014 | 0.003 | -5.695 | 0.000 |
| Mesoregion4 | -0.010 | 0.003 | -3.893 | 0.000 |
| Mesoregion5 | -0.014 | 0.002 | -5.546 | 0.000 |
| Mesoregion6 | -0.017 | 0.002 | -7.438 | 0.000 |
| Mesoregion7 | -0.011 | 0.003 | -4.382 | 0.000 |
| Mesoregion8 | -0.015 | 0.002 | -6.430 | 0.000 |
| Spring | 0.006 | 0.001 | 6.060 | 0.000 |
| Winter | 0.004 | 0.001 | 3.228 | 0.001 |

Residual standard error: 0.01735793

Parameter estimate Phi = 0.1872445

AIC = -2552.009; BIC= -2378.181; logLik= 1320.005

- *Diversity*

First model with diversity of all seabird species and Mesoregion and Season (GLS, diversity ~ Mesoregion + Season) showed a significant difference of mesoregion and season for all ecological indices (mesoregion: p<0.001. F-value = 58.0845; season: p<0.0001. F-value= 40.1556).

**S10 Table. GLS for seabird stranding diversity.** Results of generalized least squares (GLS) regression model of seabird stranding diversity spatial and temporal differences along the Brazilian coast. All variables with statistical significance are marked with an asterisk. (Reference level: mesoregion 1 in autumn)

| Parameter | Value | Std.Error | t-value | p-value |
| --- | --- | --- | --- | --- |
| (Intercept) | 0.070 | 0.004 | 17.781 | 0.000 |
| Mesoregion3 | -0.017 | 0.005 | -3.441 | 0.001 |
| Mesoregion4 | -0.016 | 0.004 | -3.626 | 0.000 |
| Mesoregion5 | -0.023 | 0.004 | -5.394 | 0.000 |
| Mesoregion6 | -0.042 | 0.004 | -10.746 | 0.000 |
| Mesoregion7 | -0.029 | 0.004 | -6.910 | 0.000 |
| Mesoregion8 | -0.037 | 0.004 | -9.179 | 0.000 |
| Spring | -0.005 | 0.002 | -2.552 | 0.011 |
| Summer | -0.010 | 0.002 | -4.760 | 0.000 |
| Winter | 0.019 | 0.003 | 6.479 | 0.000 |

Residual standard error: 0.02622038

Parameter estimate Phi = 0.1026887

AIC = -2012.618; BIC= -1838.79; logLik= 1050.309

Similarly, three other models were conducted with the response variable been (1) Diversity of Resident seabirds, (2) Diversity of Southern seabirds, (3) Diversity of Northern seabirds, in order to understand the effects of this different behaviours on the overall seabird strandings.

1. Diversity of Resident seabirds (GLS, Resident diversity ~ Mesoregion * Season) showed a significant difference of mesoregion and season for all ecological indices (mesoregion: p<0.001, F-value = 104.810; season: p= 0.6213, F-value= 0.591; mesoregion*season: p<0.001; F-value= 4.137).

**S11 Table. GLS for resident seabird stranding diversity.** Results of generalized least squares (GLS) regression model of resident seabird stranding diversity spatial and temporal differences along the Brazilian coast. Only variables with statistical significance were held here. (Reference level: mesoregion 1*autumn)

| Parameter | Value | Std.Error | t-value | p-value |
| --- | --- | --- | --- | --- |
| (Intercept) | 0,029 | 0,002 | 14,283 | 0,000 |
| Meso2 | 0,010 | 0,004 | 2,802 | 0,005 |
| Meso6 | -0,015 | 0,003 | -5,785 | 0,000 |
| Meso7 | -0,007 | 0,004 | -2,027 | 0,043 |
| Meso8 | -0,008 | 0,003 | -2,730 | 0,007 |
| Meso3:SeasonWinter | 0,016 | 0,007 | 2,387 | 0,018 |

Residual standard error: 0.006460915

Parameter estimate Phi = 0.1112608

AIC = -2661.283; BIC= -2404.491; logLik= 1395.642

1. Diversity of Southern seabirds (GLS, Southern diversity ~ Mesoregion + Season) showed a significant difference of mesoregion and season for all ecological indices (mesoregion: p<0.001, F-value = 43.3269; season: p<0.0001, F-value= 67.3372).

**S12 Table. GLS for southern seabird stranding diversity.** Results of generalized least squares (GLS) regression model of southern seabird stranding diversity spatial and temporal differences along the Brazilian coast. Only variables with statistical significance were held here. (Reference level: mesoregion 1and autumn)

| Parameter | Value | Std.Error | t-value | p-value |
| --- | --- | --- | --- | --- |
| (Intercept) | 0.043 | 0.004 | 10.959 | 0.000 |
| Meso3 | -0.025 | 0.004 | -6.231 | 0.000 |
| Meso4 | -0.021 | 0.004 | -4.915 | 0.000 |
| Meso5 | -0.022 | 0.004 | -5.201 | 0.000 |
| Meso6 | -0.029 | 0.004 | -7.662 | 0.000 |
| Meso7 | -0.030 | 0.004 | -7.642 | 0.000 |
| Meso8 | -0.031 | 0.004 | -8.279 | 0.000 |
| SeasonSummer | -0.011 | 0.002 | -5.778 | 0.000 |
| SeasonWinter | 0.013 | 0.002 | 5.375 | 0.000 |

Residual standard error 0.01795381

Parameter estimate Phi = 0.1114156

AIC = -2155.667; BIC= -1981.838; logLik= 1121.833

1. Diversity of Northern seabirds (GLS, Northern diversity ~ Mesoregion + Season) showed a significant difference of mesoregion and season for all ecological indices (mesoregion: p<0.001, F-value = 93.959; season: p<0.0001, F-value= 7.881).

**S13 Table. GLS for northern seabird stranding diversity.** Results of generalized least squares (GLS) regression model of northern seabird stranding diversity spatial and temporal differences along the Brazilian coast. Only variables with statistical significance were held here. (Reference level: mesoregion 1and autumn)

| Parameter | Value | Std.Error | t-value | p-value |
| --- | --- | --- | --- | --- |
| (Intercept) | 0.034 | 0.001 | 28.411 | 0.000 |
| Mesoregion2 | -0.004 | 0.002 | -2.001 | 0.046 |
| Mesoregion3 | -0.016 | 0.001 | -11.096 | 0.000 |
| Mesoregion4 | -0.014 | 0.002 | -8.996 | 0.000 |
| Mesoregion5 | -0.017 | 0.001 | -11.916 | 0.000 |
| Mesoregion6 | -0.025 | 0.001 | -21.601 | 0.000 |
| Mesoregion7 | -0.015 | 0.001 | -11.175 | 0.000 |
| Mesoregion8 | -0.019 | 0.001 | -14.551 | 0.000 |
| Spring | 0.003 | 0.001 | 4.728 | 0.000 |

Residual standard error: 0.01641492

Parameter estimate Phi = 0.1855442

AIC = -2810.152; BIC= -2636.324; logLik= 1449.076

- *Environmental and anthropogenic variables*

To understand how anthropogenic and environmental variables affected the observed stranding pattern of seabirds for abundance, richness and diversity, we built different models GLS with same corrections described above for variance and spatial autocorrelation but including environmental and anthropogenic variables as predictor variables. Only significant predictors were kept in the final model described below.

**S14 Table. GLS for seabird stranding in relation to environmental predictors.** Results of generalized least squares (GLS) regression model relation between all community of seabird species stranding abundance, richness and diversity and environmental predictors.

| Parameter | Value | Std.Error | t-value | p-value |
| --- | --- | --- | --- | --- |
| **Abundance** |  |  |  |  |
| (Intercept) | -0.148 | 0.092 | -1.614 | 0.107 |
| Wave Height | 0.049 | 0.014 | 3.440 | 0.001 |
| Sea surface temperature | -0.005 | 0.002 | -2.095 | 0.037 |
| Wind intensity | 0.137 | 0.024 | 5.822 | 0.000 |
|  |  |  |  |  |
| **Richness** |  |  |  |  |
| (Intercept) | 0.539 | 0.015 | 3.453 | 0.000 |
| Wave Height | 0.014 | 0.002 | 6.139 | 0.000 |
| Sea surface temperature | -0.002 | 0.000 | -5.072 | 0.000 |
| Concentration chlorophyll | 0.002 | 0.001 | 2.078 | 0.038 |
| Wind intensity | 0.001 | 0.004 | 2.590 | 0.000 |
|  |  |  |  |  |
| **Diversity** |  |  |  |  |
| (Intercept) | 0.052 | 0.007 | 7.702 | 0.000 |
| Sea surface temperature | -0.001 | 0.000 | -3.203 | 0.002 |
| Chlorophyll concentration | 0.001 | 0.000 | 2.524 | 0.012 |

**S15 Table. GLS for resident seabird stranding in relation to environmental predictors.** Results of generalized least squares (GLS) regression model relation between resident seabird species stranding abundance, richness and diversity and environmental predictors.

| Parameter | Value | Std.Error | t-value | p-value |
| --- | --- | --- | --- | --- |
| **Abundance** |  |  |  |  |
| (Intercept) | -0.109 | 0.034 | -3.23 | 0.001 |
| Wind intensity | 0.082 | 0.011 | 6.906 | 0.000 |
|  |  |  |  |  |
| **Richness** |  |  |  |  |
| (Intercept) | 0.016 | 0.004 | 3.969 | 0.000 |
| Wind intensity | 0.004 | 0.001 | 3.350 | 0.000 |
|  |  |  |  |  |

Table 16 shows the result of the model with the lowest AIC, in which only the wind intensity was maintained, but even so without presenting significance.

**S16 Table. GLS for resident seabird stranding diversity in relation to environmental predictors.** Results of generalized least squares (GLS) regression model with de lowest AIC for resident seabird species stranding diversity and environmental predictors.

|  | Value | Std.Error | t-value | p-value |
| --- | --- | --- | --- | --- |
| Intercepto | 0.020 | 0.002 | 8.249 | 0.000 |
| Wind intensity (m/s) | 0.001 | 0.001 | 1.743 | 0.082 |

Residual standard error: 0.01198348

Parameter estimate Phi = 0.7174714

AIC = -2534.736; BIC= -2396.463; logLik= 1302.368

**S17 Table. GLS for southern seabird stranding in relation to environmental predictors.** Results of generalized least squares (GLS) regression model relation between southern seabird species stranding abundance, richness and diversity and environmental predictors.

| Parameter | Value | Std.Error | t-value | p-value |
| --- | --- | --- | --- | --- |
| **Abundance** |  |  |  |  |
| (Intercept) | 0.167 | 0.023 | 7.101 | 0.000 |
| Sea surface temperature | -0.006 | 0.001 | -6.858 | 0.000 |
|  |  |  |  |  |
| **Richness** |  |  |  |  |
| (Intercept) | 0.108 | 0.011 | 10.113 | 0.000 |
| Sea surface temperature | -0.004 | 0.000 | -13.507 | 0.000 |
| Wind intensity | 0.005 | 0.002 | 3.098 | 0.002 |
|  |  |  |  |  |
| **Diversity** |  |  |  |  |
| (Intercept) | 0.088 | 0.008 | 10.483 | 0.000 |
| Sea surface temperature | -0.004 | 0.000 | -14.075 | 0.000 |
| Wind intensity | 0.005 | 0.001 | 4.042 | 0.000 |

**S18 Table. GLS for northern seabird stranding in relation to environmental predictors.** Results of generalized least squares (GLS) regression model relation between northern seabird species stranding abundance, richness and diversity and environmental predictors.

| Parameter | Value | Std.Error | t-value | p-value |
| --- | --- | --- | --- | --- |
| **Abundance** |  |  |  |  |
| (Intercept) | 0.008 | 0.005 | 1.542 | 0.124 |
| Wave height | 0.009 | 0.003 | 3.645 | 0.000 |
| Chlorophyll concentration | 0.002 | 0.001 | 2.292 | 0.023 |
|  |  |  |  |  |
| **Richness** |  |  |  |  |
| (Intercept) | -0.008 | 0.003 | -2.984 | 0.000 |
| Wave height | 0.002 | 0.001 | 3.537 | 0.000 |
| Wind intensity | 0.005 | 0.001 | 5.283 | 0.000 |
|  |  |  |  |  |
| **Diversity** |  |  |  |  |
| (Intercept) | -0.002 | 0.002 | -0.815 | 0.416 |
| Wave height | 0.001 | 0.000 | 3.267 | 0.001 |
| Sea surface temperature | 0.000 | 0.000 | 2.197 | 0.029 |
| Wind intensity | 0.003 | 0.001 | 5.177 | 0.000 |

**Bibliography**

1. Somenzari M, do Amaral PP, Cueto VR, Guaraldo A de C, Jahn AE, Lima DM, et al. An overview of migratory birds in Brazil. Pap Avulsos Zool. 2018;58. doi:10.11606/1807-0205/2018.58.03
2. Pacheco JF, Silveira LF, Aleixo A, Agne CE, Bencke GA, Bravo GA, et al. Annotated checklist of the birds of Brazil by the Brazilian Ornithological Records Committee—second edition. Ornithology Research. 2021;29: 94–105. doi:10.1007/s43388-021-00058-x
3. Billerman SM, Keeney BK, Rodewald PG, Schulenberg TS, editors. Birds of the World. https://birdsoftheworld.org/bow/home. Ithaca, NY, USA: Cornell Laboratory of Ornithology; 2022. Available: <https://birdsoftheworld.org/bow/home>
